# Prevalence and Determinants of Hoarseness in School-Aged Children

**DOI:** 10.3390/ijerph19095468

**Published:** 2022-04-30

**Authors:** Ahmed Alrahim, Askar K. Alshaibani, Saad Algarni, Abdulmalik Alsaied, Amal A. Alghamdi, Salma Alsharhan, Mohammad Al-Bar

**Affiliations:** 1 ENT Department, King Fahad Hospital of the University, College of Medicine, Imam Abdulrahman bin Faisal University, Dammam 34212, Saudi Arabia; assaied@iau.edu.sa (A.A.); ssalsharhan@iau.edu.sa (S.A.); malbar@iau.edu.sa (M.A.-B.); 2 College of Medicine, Imam Abdulrahman bin Faisal University, Dammam 34212, Saudi Arabia; akalshaibani@iau.edu.sa (A.K.A.); sasalgarni@iau.edu.sa (S.A.); 3 Department of Family and Community Medicine, College of Medicine, Imam Abdulrahman bin Faisal University, Dammam 34212, Saudi Arabia; amlalghamdi@iau.edu.sa

**Keywords:** hoarseness, voice disorders, school age, children

## Abstract

Hoarseness in school-aged children may affect their educational achievement and interfere with their communication and social skills development. The global prevalence of hoarseness in school-aged children ranges between 6% and 23%. To the best of our knowledge, there is a scarcity of studies describing the prevalence or determinates of hoarseness in Saudi school-aged children. Our aim was to measure the prevalence of hoarseness among school-aged children and to identify its determinants. A cross-sectional questionnaire-based survey was used that included randomly selected primary and early childhood schools from private and governmental sectors in Saudi Arabia. The data were collected using a questionnaire which was self-completed by the children’s parents and covered the following aspects: sociodemographic features, health and its related comorbidities about children and their families, attendance and performance in school, child’s voice tone, past history of frequent crying during infancy, history of letter pronunciation problems and stuttering, the Reflux Symptom Index (RSI) and the Children’s Voice Handicap Index-10 for parents (CVHI-10-P). Determinants of hoarseness were investigated using the SPSS software (version 20). The mean age of the study children (*n* = 428) was 9.05 years (SD = 2.15), and 69.40% of them were male. The rate of hoarseness in the participants was 7.5%. Hoarseness was significantly common in children with a history of excessive infancy crying (*p* = 0.006), letter pronunciation issues (especially ‘R’ and ‘S’; *p* = 0.003), and stuttering (*p* = 0.004) and in those with a previous history of hoarseness (*p* = 0.023). In addition, having the symptoms of gastrointestinal reflux increased the risk of hoarseness by four times (OR = 4.77, 95% CI = 2.171, 10.51). In summary, hoarseness in children may be dangerously underestimated, as it may reflect the presence of speech problems, in addition to the presence of laryngopharyngeal reflux (LPR). Hoarseness was assumed on the basis of parental complaints. Therefore, further research with diagnoses based on a clinical assessment is needed to understand the magnitude of the hoarseness problem and its consequences in children.

## 1. Introduction

Hoarseness or dysphonia is characterized by altered vocal quality, pitch, loudness, or vocal effort that impairs communication or reduces voice-related quality of life (QOL) [1]. It can affect all age groups and all genders, with a prevalence of approximately 29.9% and is more common among professional voice users such as singers or teachers [2,3]. Children are also susceptible to develop hoarseness, with a prevalence ranging from 6% to 23% among school-aged children [4]. It has been estimated that 2–4% of children with voice problems have never consulted a speech language pathologist for the management of these issues [5]. Voice disorders in children might negatively affect their personality and educational progress, which can eventually affect their normal developmental milestones [6]. Thus, it is important to address and manage this problem as early as possible. In adults, the prevalence of hoarseness is higher in females than in males [3]. However, the opposite has been observed in children, with a higher prevalence among males than in females, due to the presence deferent pathologies more commonly in males such as vocal nodules, subglottic stenosis and croup, while no gender difference was observed in children with hoarseness and a normal larynx between the genders [7]. There are many different factors that lead to hoarseness, such as personal, environmental, psychosocial and genetic factors [8,9]. Depending on the etiology, voice disorders in children can be treated medically, surgically or through voice therapy [6].

The Voice Handicap index (VHI) is one of the most common validated tools used by clinicians to subjectively measure multiple aspects of voice disorders including physical, functional, and emotional aspects [10].

The VHI was developed by Jacobson et al. in 1997, with a set of 30 items; in 2004, a simplified, less time-consuming and easier-to-use version of a 10-item questionnaire was developed by Rosen et al. and it has been adopted and translated into many languages [11,12]. The VHI is mainly used for adult patients and cannot be used for children as it is difficult for them to understand it. Therefore, many different tools, such as the Pediatric Voice Handicap Index (pVHI), the Children’s Voice Handicap Index-10 (CVHI-10), and the Children’s Voice Handicap Index-10 for Parents (CVHI-10-P), were developed from the VHI and were validated for use in children [13,14,15]. The CVHI-10-P is a set of validated 10 items used to assess parents’ perspective toward their children’s voice to help clinicians to better assess voice disorders in children.

The Reflux Symptom Index (RSI) is one of the tools available to subjectively assess the laryngopharyngeal reflux (LPR) severity that was developed and validated by Belafsky in 2002 [16]. It has been found that up to 50% of adult patients with voice disorders also have LPR symptoms [17]. Block et al. found that 47% of children who presented with the main complaint of hoarseness were diagnosed with LPR [18]. This study aimed to measure the prevalence of hoarseness among school-aged children and to find its possible determinant factors.

## 2. Methodology

This was a cross-sectional questionnaire-based survey conducted randomly in 21 early childhood schools and primary schools in the private and government sectors from April 2019 till November 2019 in the Eastern Province of Saudi Arabia. Three cities were chosen in the eastern province of Saudi Arabia: Dammam, Alkhobar and Aljubail. In each city, around 7 schools were chosen randomly. The selection was carried out through blind random draws from the list of school in each city.

Teachers at the chosen schools were contacted to send a questionnaire to the parents of the students within their school using the class Email group. The questionnaire was answered by 428 parents of a child, either by the father or by the mother, attending one of the randomly chosen schools. The data collection lasted for a month, and the response rate was around 50%. The survey was designed on the basis of previously published literature as well as our clinical observations [15,16,19].

The questionnaire was completed by one of the parents of the chosen class children and covered the following aspects: sociodemographic features, health and its related comorbidities and medications, household pets (presence vs. absence), number of children in the family and child birth order (first vs. middle vs. last), number of classes the child attended per day, number of students per class, missing days from schools due to voice problems (yes vs. no), usual degree of child’s voice tone (low vs. moderate vs. high), child’s participation in school activities or sport exercises that require a loud voice (yes vs. no), family history of voice disorders (yes vs. no), past history of frequent excessive crying during infancy (yes vs. no), history of letter pronunciation problems (yes vs. no) and stuttering (yes vs. no), history of intubation (yes vs. no), history of having a cold or recurrent cough the past 2 years (yes vs. no), history of hearing problems (yes vs. no), parent smoking status (smoker vs. non-smoker vs. ex-smoker), history of vocal fold surgery (yes vs. no), Reflux Symptom Index (RSI) [16], and Children’s Voice Handicap Index-10 for parents (CVHI-10-P) [15]. CVHI-10-P is a self-assessment tool used to assess parents’ perspective of their child’s voice handicap. It is a set of 10 questions assessing different aspects of a child’s life including physical, functional and emotional aspects. Each question is scored based on parents’ answer as 0 = never, 1 = sometime, 2 = many time, 3 = always. A CVHI-10-P score > 11 indicates a subjective diagnosis of voice disorders. RSI is a self-assessment tool used to assess LPR symptoms and severity. It is a set of 9 questions focusing on reflex symptoms that commonly affect patients. Each question is measured into a scale from 0 = no problem to 5 = severe problem. An RSI with a score > 13 indicates a subjective diagnosis of LPR.

This study was approved by the Institutional Review Board (IRB) of King Fahd hospital (IRB-2019-01-271), and the parents’ consent was given in advance and at the end of the questionnaire. We attached an optional question for children with voice disorders who want to seek medical care in our otolaryngology department for further management.

Data were entered, and factors associated with hoarseness were investigated and analyzed using the Chi-square test, Fisher’s exact test, and adjusted and unadjusted logistic regression models via the SPSS software version 20 (IBM, Chicago, IL, USA).

For statistical analysis, *p* < 0.05 was considered statistically significant.

## 3. Results

The total number of participants was 428, and the majority of them (90.9%, *n* = 389) were in primary school, whilst the rest were in kindergarten (9.1%, *n* = 39). The mean age of the participants was 9.05 years (standard deviation (SD) = 2.15); 69.40% (*n* = 297) of the participants were males, and 30.6% (*n* = 131) were females. Almost 60% of the participants (*n* = 260) had two to four siblings, and 34% (*n* = 147) had more than five siblings. Half of the children were a middle child, and (50.9%, *n* = 218) and third of them were firstborns (33.2%, *n* = 142), whilst the majority of the children lived with both their parents (93.2%, *n* = 399), Table 1. The prevalence of hoarseness among our participants was 7.5%, as 9.90% of female and 6.40% of male children had hoarseness. Eighty-five percent (*n* = 366) of the participants frequently used a medium to high pitch voice tone

In regard to the voice problem indicators measured using the Children’s Voice Handicap Index-10 for parents, approximately 4.2% (*n* = 18) of the parents reported that their children had severe hoarseness, and 19.9% (*n* = 85) of them reported that their children’s voice was difficult to hear, whilst 13.3% (*n* = 57) of them claimed that their children’s voice reduced their school outcome, and 11.2% (*n* = 48) of the parents claimed that their children’s voice made them feel inferior to other children, Table 2.

As seen in Table 3 and Table 4, the rate and risk of hoarseness were statistically significantly higher in children with a history of excessive infancy crying (odd’s ratio (OR) = 2.733, 95% confidence interval (CI) = 1.315 to 5.683)), a previous history of hoarseness (OR = 2.315, 95% CI = 1.109 to 4.832), letter articulation problems (especially ‘R’ and ‘S’; OR = 2.969, 95% CI = 1.421 to 6.204), stuttering (OR = 3.213, 95% CI = 1.428 to 7.228), a higher number of classes the child attended per day (OR = 0.0320, 95% CI = 0.145 to 0.703). In addition, only two children had previous vocal cord surgery, one of whom had hoarseness.

In regard to the RSI, the average score of all participants was 5.08 (SD = 6.34, minimum = 0, maximum = 40), and the number of participants with an RSI > 13 was 55 (12.9%). In addition, it was found that the risk of hoarseness increased by four times if the RSI score was >13 (OR = 4.77, 95% CI = 2.17, 10.51).

## 4. Discussions

This study aimed to measure the rate of hoarseness among school-aged children. In our study, we found that the prevalence of hoarseness was 7.5%, in accordance with the range reported in the literature 6–23% [4]. Dobres et al. found in their study on the description of laryngeal pathologies in children with a sample size of 731 patients that the prevalence was higher in males than in females, similar to the findings of Kallvik et al. in their study on the prevalence of hoarseness in school-aged children with a sample size of 217 participants [7,19]. Children have a lesser amount of elastin, and their vocal fold is less stable than that of adults, who have a significant amount of elastin and collagen which provide more stability and the elastic proprieties of the vocal folds. Consequently, the vocal fold of children with recurrent voice misuse vibrates more forcefully because of a lack of elastic proprieties, increasing their risk of injury compared to adults [20,21,22]. These observations may explain the significant rate of hoarseness in children.

In our study, there was no significant difference with respect to gender. It is suggested that the impact of gender on the voice is not significant in early childhood until the period when each gender acquires his or her specific voice tone and pattern as well as the social specific behavior of each gender [19,23,24].

Concerning the possible risk factors of hoarseness, we found that a history of excessive crying during infancy was a possible associated factor of current hoarseness in our study. Kallvik et al. reported a similar finding of a history of heavy voice use during infancy being associated significantly with the current voice quality, especially among females [19]. Moreover, we found that a previous history of hoarseness was a factor significantly associated with current hoarseness. It might be explained that the continued voice misuse in children and a lack of education through voice therapy might keep them at high risk of repeated voice disorders.

In our study, we found that letter articulation problems or what is known as phonation disorder was an associated factor of hoarseness in children. The exact reason is unknown, but this might be due to the fact that a child with a problem in letter articulation will try hard to articulate the letters correctly, putting his voice in a repeated tension, along with the misuse of his voice, eventually leading to voice disorders.

Additionally, in our study we found that stuttering was another possible risk factor for hoarseness. Stutterer will produce repeated words, sounds, sentences or sudden involuntary breaks which might lead to abnormal physical and emotional behaviors as the speaker struggles to end a particular sentence [25]. Stuttering could be developmental, which is the most common types, or acquired secondary to brain injury or emotional trauma [26,27]. Salihović et al. studied the voice characteristics in stuttering children in their case–control experimental study and found that the abnormal functioning of the larynx and high muscular tension as well as subglottic pressure with lack of coordination and control of the respiratory and laryngeal muscles will lead to voice disorders [28]. Children with stuttering were found to be at high risk of developing social anxiety, low self-esteem and a decreased quality of life in the future [29,30]. In our study, the parents of children with hoarseness reported that their children educational outcome was affected by hoarseness; additionally, they reported that their children were feeling inferior to other children and felt bothered by their voices. In this regard, an early diagnosis of stuttering is important, as it was found to have a very promising outcome with early interventions [31].

The LPR is an inflammatory reaction caused by the backflow of gastric acid, which leads to laryngitis and pharyngitis [32]. We found that those having symptoms suggesting LPR (RSI > 13) had a four-fold increased risk of hoarseness. Block et al. found in their study on the role of LPR in children with hoarseness, with sample size of 337 participants, that 47% of children who presented with the main complaint of hoarseness were diagnosed with LPR, and 68% of patients showed an improvement in hoarseness after initiating an anti-reflux therapy [18]. Moreover, Gumpert et al. found in their study that 90% of children with hoarseness had endoscopic signs of LPR [33]. Carr et al. described the endoscopic findings of LPR in their study as lingual tonsillar hyperplasia, postglottic edema and arytenoid edema [34]. Remarkably, the study of Block et al. found that 48% of patients diagnosed with LPR had no cough or throat clearing, which are the most common symptoms seen in children with LPR. Hence, hoarseness in children is an important key for diagnosing LPR [18]. Most studies in the literature correlate the endoscopic finding of LPR with subjective complaints of hoarseness, i.e., there is no acoustic voice analysis for hoarseness with an objective assessment. However, Niedzielska et al. conducted a study on 11 participants in whom reflux disease was confirmed with pH-metric assessment in the esophagus as well as objective voice measurements with lupolaryngoscopy, stroboscopy and acoustic voice analysis (jitter, shimmer, harmonic/noise ratio and phonation time) [35]. They confirmed the correlation between an inflammatory change in the larynx and voice disorders.

As far as our knowledge goes, our study is the first large-scale study that examined hoarseness in school-age children in Saudi Arabia and investigated its related risk factors. However, our study has some limitations, including the subjective nature of the used tools (RSI and CVHI-10-P) and the lack of appropriate objective clinical and endoscopic evaluations of the larynx and head and neck region to confirm the diagnosis. Furthermore, one cannot solely depend on RSI for the diagnosis of LPR, and more appropriate diagnostic tools, such as an assessment of the patient’s pH levels, are needed [36]. However, clinical assessment was offered to children of interested parents for further evaluation for their children voices, but the resultant findings were not included in the current study. Using a cross-sectional design limited the ability to identify the temporal sequences of events; thus, causality of the identified risk factors could not be established, especially considering the retrospective nature of the questionnaire and the possibility of recall bias and measurement errors. However, due to the aim of examining a large sample of schools (*n* = 21) in the eastern province of the kingdom, parents were reached by their child’s class teacher via an online self-completed questionnaire which, unfortunately, resulted in a limited response rate from the parents and, consequently, a possibility of selection bias. However, the resulted prevalence and risk estimations of factors associated with hoarseness in our study were comparable to those reported in the literature; therefore our results can be trusted.

In fact, the limited response rate might indicate the possibility of a low level of parents’ awareness regarding voice disorders in children and the need of further parent education to raise their awareness in the future.

## 5. Conclusions

Hoarseness in children may not be common but can be dangerously underestimated, as it may reflect the presence of speech problems in addition to the presence of laryngopharyngeal reflux (LPR). A history of excessive crying in infancy, letter pronunciation issues, stuttering, a previous history of hoarseness and having the symptoms of gastrointestinal reflux increase the risk of hoarseness in children.

Hoarseness was assumed on the basis of parental complaints. Therefore, further research with diagnoses based on a clinical assessment is needed to understand the magnitude of the hoarseness problem and its consequences in children.

## Figures and Tables

**Table 1:** Distribution of participants’ sociodemographic data (*n* = 428).

		Total Number	Without Hoarseness	With Hoarseness	Chi-Square *p* Value
Age	≤5 years	28 (6.5%)	26	2	0.974
	6–9	179 (41.8%)	165	14	
	≥10	221 (51.6%)	205	16	
Gender	male	297 (69.4%)	278	19	0.201
	female	131 (30.6%)	118	13	
Number of siblings	1	21 (4.9%)	19	2	0.5
	2–4	260 (60.7%)	247	13	
	≥5	147 (34.3%)	130	17	
Child’s birth order	1	142 (33.2%)	134	8	0.131
	2–4	218 (50.9%)	203	15	
	≥5	68 (15.9%)	59	9	
Child lives with	both parents	399 (93.2%)	368	31	0.659
	one parent	24 (5.6%)	23	1	
	relatives	5 (1.2%)	5	0	
Level of education	kindergarten	39 (9.1%)	35	4	0.489
	primary school	389 (90.9%)	361	28	

**Table 2:** Distribution of voice hoarseness indicators measured using the Children’s Voice Handicap Index-10 for parents.

Items	Categories *	*n*	%
Voice tone	screaming	37	8.6
	high	139	32.5
	medium	227	53.0
	low	25	5.8
How severe is the hoarseness	severe	18	4.2
	moderate	120	28.0
	mild	117	27.3
Which part of the day does hoarseness occur	morning	102	23.8
	noon	28	6.5
	afternoon	69	16.1
	night	38	8.9
People have difficulty hearing my child because of his voice	no	343	80.1
	yes	85	19.9
People have difficulty understanding my child in a noisy room	no	287	67.1
	yes	141	32.9
The voice difficulties of my child prevent him to stay with people	no	384	89.7
	yes	44	10.3
My child feels left out of conversations because of his voice	no	390	91.1
	yes	38	8.9
The voice difficulties of my child reduce his school outcome	no	371	86.7
	yes	57	13.3
My child feels he has to strain to produce voice	no	350	81.8
	yes	78	18.2
The voice of my child is not light	no	231	54.0
	yes	197	46.0
The voice problem of my child upsets him	no	342	79.9
	yes	86	20.1
The voice of my child makes him feel inferior to other children or other boys	no	380	88.8
	yes	48	11.2
People ask me ‘‘what’s wrong with the voice of your child?”	no	356	83.2
	yes	72	16.8

* Yes indicates those who answered the question with 1 (sometime), 2 (many time), or 3 (always), combined together as yes. * No indicates those who answered the question with 0 (never), indicated as no.

**Table 3:** Distribution of risk factors of hoarseness and estimated risk of hoarseness in relation to these risk factors.

		Total	Without Hoarseness		With Hoarseness		Chi-Square Test	
		*n*	*n*	%	*n*	%	*X*^2^ (1)	*p*
Household pets	no	382	351	91.88	31	8.12	2.10	0.148
	yes	46	45	97.83	1	2.27		
Previous history of hoarseness	no	255	242	94.90	13	5.10	5.16	0.023
	yes	173	154	89.02	19	10.98		
Parents had hoarseness	no	261	244	93.45	17	6.51	0.90	0.344
	yes	167	152	91.01	15	9.98		
Cried excessively in infancy	no	282	268	95.04	14	4.96	7.54	0.006
	yes	146	128	87.07	18	12.34		
Exercise that requires a loud voice	no	344	318	92.44	26	7.56	0.02	0.897
	yes	84	78	92.86	6	7.14		
Pronunciation issues	no	310	294	94.84	16	5.16	8.71	0.003
	yes	118	102	86.44	16	13.56		
Stuttering in the last year	no	367	343	93.97	22	6.03	8.08	0.004
	yes	61	51	83.61	10	16.39		
Having a cold in the past 2 years	non frequent	379	353	93.14	25	6.59	1.82	0.177
	frequent	49	43	87.75	6	12.24		
Having cough in the past 2 years	non frequent	386	358	92.75	28	7.25	0.28	0.595
	frequent	42	38	90.47	4	9.52		
Previous intubation	no	413	384	92.98	29	7.02		0.093
	yes	15	12	80.00	3	20.00		
Allergy	no	308	281	91.23	27	8.76	2.64	0.104
	yes	120	115	95.83	5	4.16		
Hearing impairment	no	397	368	92.70	29	7.30	0.23	0.629
	yes	31	28	90.32	3	9.68		
Asthma	no	386	356	92.23	30	7.73		0.757
	yes	42	40	95.24	2	4.76		
Parents are smokers or previous smokers	no	240	226	94.16	14	5.83	2.13	0.144
	yes	188	170	90.43	18	9.57		
RSI score > 13	no	373	353	94.60	20	5.40	18.76	<0.001
	yes	55	43	78.20	12	21.80		

(*n*)= number, (*p*) = *p*-value < 0.05, (*X*^2^) = chi-square test.

**Table 4:** Distribution of risk factors of hoarseness and unadjusted and adjusted regressions of hoarseness in relation to these risk factors.

	Total		Unadjustd Regression			Adjusted Regression *
		*n*	OR	95% CI	OR	95% CI
Household pets	no	382	Ref.			
	yes	46	0.25	0.03, 1.89	0.25	0.03, 1.89
Previous history of hoarseness	no	255	Ref.			
	yes	173	2.30	1.10, 4.78	22.32	1.11, 4.83
Parents had hoarseness	no	261	Ref.			
	yes	167	1.42	0.69, 2.92	1.37	0.67, 2.83
Cried excessively in infancy	no	282	Ref.			
	yes	147	2.69	1.30, 5.58	2.73	1.32, 5.68
Exercise that requires a loud voice	no	344	Ref.			
	yes	84	0.94	0.37, 2.37	0.95	0.38, 2,40
Pronunciation issues	no	310	Ref.			
	yes	118	2.89	1.39, 5.97	2.97	1.42, 6.20
Stuttering in the last year	no	365	Ref.			
	yes	61	3.06	1.37, 6.82	3.21	1.43, 7.23
Having a cold in the past 2 years	non frequent	379	Ref.			
	frequent	49	0.53	0.21, 136	0.52	0.20, 1.34
Having cough in the past 2 years	non frequent	386	Ref.			
	frequent	42	0.74	0.25, 2.23	0.73	0.24, 2.19
Previous intubation	no	413	Ref.			
	yes	15	3.31	0.88	3.29	0.87, 12.37
Allergy	no	308	Ref.			
	yes	120	0.45	0.17, 1.20	0.46	0.17, 1.22
Hearing impairment	no	397	Ref.			
	yes	31	1.36	0.39, 4.74	1.38	0.39, 4.85
Asthma	no	386	Ref.			
	yes	42	0.59	0.14, 2.58	0.63	0.15, 2.76
Parents are smokers or previous smokers	no	240	Ref.			
	yes	188	1.71	0.83, 3.53	1.79	0.86, 3.71
RSI score > 13	no	373	Ref.			
	yes	55	4.93	2.25, 10.77	4.78	2.17, 10.51

* adjusted for age and gender. (OR) = odd ratio, (CI) = confidence interval, (*n*) = number.

## Data Availability

The data presented in this study are available on request from the corresponding author. The data are not publicly available due to ethical and privacy reasons.

## References

[1] Schwartz S.R., Cohen S., Dailey S.H., Rosenfeld R.M., Deutsch E.S., Gillespie M.B., Granieri E., Hapner E., Kimball C.E., Krouse H.J. (2009). Clinical practice guideline: Hoarseness (dysphonia). Otolaryngol. Head Neck Surg..

[2] Roy N., Merrill R.M., Thibeault S., Parsa R.A., Gray S.D., Smith E.M. (2004). Prevalence of voice disorders in teachers and the general population. J. Speech Lang. Heart Res..

[3] Roy N., Merrill R.M., Gray S.D., Smith E.M. (2005). Voice disorders in the general population: Prevalence, risk factors, and occupational impact. Laryngoscope.

[4] Maddern B.R., Campbell T.F., Stool S. (1991). Pediatric voice disorders. Otolaryngol. Clin. N. Am..

[5] Kahane J.C., Mayo R. (1989). The need for aggressive pursuit of healthy childhood voices. Lang. Speech Heart Serv. Sch..

[6] Verduyckt I., Remacle M., Jamart J., Benderitter C., Morsomme D. (2011). Voice-related complaints in the pediatric population. J. Voice.

[7] Dobres R., Lee L., Stemple J.C., Kummer A.W., Kretschmer L.W. (1990). Description of laryngeal pathologies in children evaluated by otolaryngologists. J. Speech Heart Disord..

[8] Vilkman E. (2004). Occupational safety and health aspects of voice and speech professions. Folia Phoniatr. Logop..

[9] Simberg S., Santtila P., Soveri A., Varjonen M., Sala E., Sandnabba N.K. (2009). Exploring genetic and environmental effects in dysphonia: A twin study. J. Speech Lang. Heart Res..

[10] Jacobson B.H., Johnson A., Grywalski C., Silbergleit A., Jacobson G.P., Benninger M.S., Newman C.W. (1997). The voice handicap index (VHI): Development and validation. Am. J. Speech-Lang. Pathol..

[11] Rosen C.A., Lee A.S., Osborne J., Zullo T., Murry T. (2004). Development and validation of the voice handicap index-10. Laryngoscope.

[12] Núñez-Batalla F., Corte-Santos P., Señaris-González B., Llorente-Pendás J.L., Górriz-Gil C., Suárez-Nieto C. (2007). Adaptación y validación del índice de incapacidad vocal (VHI-30) y su versión abreviada (VHI-10) al español. Acta Otorrinolaringol. Esp..

[13] Zur K.B., Cotton S., Kelchner L., Baker S., Weinrich B., Lee L. (2007). Pediatric Voice Handicap Index (pVHI): A new tool for evaluating pediatric dysphonia. Int. J. Pediatr. Otorhinolaryngol..

[14] Ricci-Maccarini A., De Maio V., Murry T., Schindler A. (2013). Development and validation of the children’s voice handicap index-10 (CVHI-10). J. Voice.

[15] Ricci-Maccarini A., De Maio V., Murry T., Schindler A. (2016). Development and validation of the children’s voice handicap index-10 for parents. J. Voice.

[16] Belafsky P.C., Postma G.N., Koufman J.A. (2002). Validity and reliability of the reflux symptom index (RSI). J. Voice.

[17] Koufman J.A., Amin M., Panetti M. (2000). Prevalence of reflux in 113 consecutive patients with laryngeal and voice disorders. Otolaryngol. Head Neck Surg..

[18] Block B.B., Brodsky L. (2007). Hoarseness in children: The role of laryngopharyngeal reflux. Int. J. Pediatr. Otorhinolaryngol..

[19] Kallvik E., Lindström E., Holmqvist S., Lindman J., Simberg S. (2015). Prevalence of hoarseness in school-aged children. J. Voice.

[20] Chan R.W., Fu M., Young L., Tirunagari N. (2007). Relative contributions of collagen and elastin to elasticity of the vocal fold under tension. Ann. Biomed. Eng..

[21] Hammond T.H., Gray S.D., Butler J., Zhou R., Hammond E. (1998). Age- and gender-related elastin distribution changes in human vocal folds. Otolaryngol. Head Neck Surg..

[22] Moore J., Thibeault S. (2012). Insights into the role of elastin in vocal fold health and disease. J. Voice.

[23] Duff M.C., Proctor A., Yairi E. (2004). Prevalence of voice disorders in African American and European American preschoolers. J. Voice.

[24] Ferrand C.T., Bloom R.L. (1996). Gender differences in children’s intonational patterns. J. Voice.

[25] Conture E.G. (2001). Stuttering: Its Nature, Assessment, and Treatment.

[26] Büchel C., Sommer M. (2004). What causes stuttering?. PLoS Biol..

[27] Theys C., van Wieringen A., Sunaert S., Thijs V., De Nil L. (2011). A one year prospective study of neurogenic stuttering following stroke: Incidence and co-occurring disorders. J. Commun. Disord..

[28] Salihović N., Junuzović-Žunić L., Ibrahimagić A., Beganović L. (2009). Characteristics of voice in stuttering children. Acta Med. Salin..

[29] Erickson S., Block S. (2013). The social and communication impact of stuttering on adolescents and their families. J. Fluen. Disord..

[30] Iverach L., Rapee R.M. (2014). Social anxiety disorder and stuttering: Current status and future directions. J. Fluen. Disord..

[31] Weir E., Bianchet S. (2004). Developmental dysfluency: Early intervention is key. Can. Med. Assoc. J..

[32] Ford C.N. (2005). Evaluation and management of laryngopharyngeal reflux. JAMA.

[33] Gumpert L., Kalach N., Dupont C., Contencin P. (1998). Hoarseness and gastroesophageal reflux in children. J. Laryngol. Otol..

[34] Carr M.M., Nagy M.L., Pizzuto M.P., Poje C.P., Brodsky L.S. (2001). Correlation of findings at direct laryngoscopy and bronchoscopy with gastroesophageal reflux disease in children: A prospective study. Arch. Otolaryngol. Head Neck Surg..

[35] Niedzielska G., Wroczek-Glijer E., Toman D. (2000). Voice disorders in children with gastroesophageal reflux disease. Otolaryngol. Pol..

[36] Katz P.O. (1990). Ambulatory esophageal and hypopharyngeal pH monitoring in patients with hoarseness. Am. J. Gastroenterol..

